# Neither too much nor too little: mitochondrial calcium concentration as a balance between physiological and pathological conditions

**DOI:** 10.3389/fmolb.2023.1336416

**Published:** 2023-12-12

**Authors:** Donato D’Angelo, Denis Vecellio Reane, Anna Raffaello

**Affiliations:** ^1^ Department of Biomedical Sciences, University of Padua, Padua, Italy; ^2^ Institute for Diabetes and Obesity, Helmholtz Zentrum München, Munich, Germany; ^3^ Department of Biomedical Sciences, Myology Center (CIR-Myo), University of Padua, Padua, Italy

**Keywords:** mitochondria, calcium, mitochondrial calcium uniporter (MCU), cell death, metabolism

## Abstract

Ca^2+^ ions serve as pleiotropic second messengers in the cell, regulating several cellular processes. Mitochondria play a fundamental role in Ca^2+^ homeostasis since mitochondrial Ca^2+^ (mitCa^2+^) is a key regulator of oxidative metabolism and cell death. MitCa^2+^ uptake is mediated by the mitochondrial Ca^2+^ uniporter complex (MCUc) localized in the inner mitochondrial membrane (IMM). MitCa^2+^ uptake stimulates the activity of three key enzymes of the Krebs cycle, thereby modulating ATP production and promoting oxidative metabolism. As Paracelsus stated, “Dosis sola facit venenum,”in pathological conditions, mitCa^2+^ overload triggers the opening of the mitochondrial permeability transition pore (mPTP), enabling the release of apoptotic factors and ultimately leading to cell death. Excessive mitCa^2+^ accumulation is also associated with a pathological increase of reactive oxygen species (ROS). In this article, we review the precise regulation and the effectors of mitCa^2+^ in physiopathological processes.

## 1 Introduction

Intracellular calcium ions (Ca^2+^) serve as a widespread second messenger, regulating a multitude of cellular functions such as gene expression, metabolism, muscle contraction, synaptic plasticity, cell proliferation, and death. The intricate control of Ca^2+^ signaling enables cell-specific control of these biological processes in space and time (Berridge, 2001). There are several sources of Ca^2+^ that cooperate to elevate the concentration of Ca^2+^ in the cytosol [(Ca^2+^)_cyt_ ∼ 100 nM]. These sources encompass Ca^2+^ from the extracellular milieu [(Ca^2+^)_ext_ ∼ 1 mM], and intracellular Ca^2+^ reservoirs, primarily the endoplasmic reticulum (ER)—recognized as the sarcoplasmic reticulum (SR) in striated muscle cells [(Ca^2+^)_ER/SR_ > 100 μM] (PMID: 8036248; PMID: 16371601). In this context, mitochondria play a pivoltal role. Indeed, in response to an increase in [Ca^2+^]_cyt_, mitochondria can uptake Ca^2+^ through a process that depends on three prerequisites. 1) The electrochemical proton gradient (ΔμH^+^), generated by the translocation of H^+^ ions across the inner mitochondrial membrane (IMM) due to the activity of the electron transport chain (ETC). It comprises the membrane potential difference (ΔΨ) and the H^+^ concentration difference (ΔpH), with ΔΨ being predominant (Mitchell and Moyle, 1967). The negative ΔΨ (∼-180 mV) represents the driving force for Ca^2+^ accumulation within the mitochondrial matrix. 2) The microdomains between ER/SR and the plasma membrane with the mitochondria. Mitochondria are closely located to ER/SR Ca^2+^ channels (inositol-1,4,5-triphosphate-receptors [Ins(1,4,5)P_3_Rs] and ryanodine receptors (RYRs), as well as plasma membrane Ca^2+^ channels (store-operated channels and voltage-operate channels). This enables mitochondria to promptly sense microdomains of high [Ca^2+^]_cyt_ (Rizzuto et al., 1998; Csordás et al., 1999; Szalai et al., 2000). 3) The presence of the mitochondrial Ca^2+^ uniporter (MCU) complex in the IMM, a sophisticated mechanism that enables Ca^2+^ entry into the mitochondrial matrix (discussed in the next paragraph).

In this review, we will provide a brief overview of the structure and function of the MCU complex. We will then focus on the regulators of mitCa^2+^. Specifically, we will discuss the role of mitochondria as cytosolic Ca^2+^ buffers and the role of mitCa^2+^ in the regulation of oxidative metabolism, cell death, and ROS production.

## 2 Structure and function of the MCU complex

**FIGURE 1:**
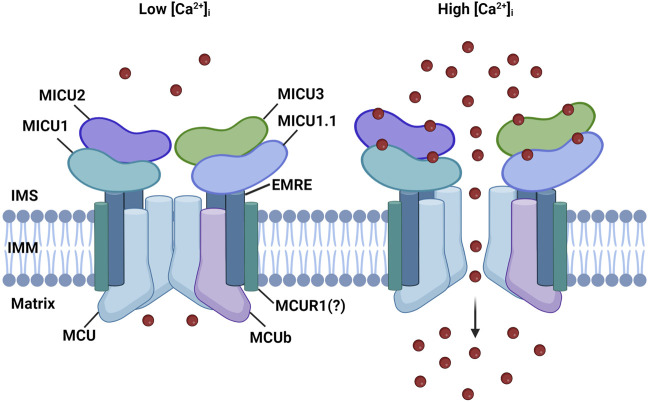
MCU complex structure. The MCU complex is localized in the IMM. It comprises the pore-forming subunit, MCU, and its dominant-negative form, MCUb It consists of the pore-forming subunit, MCU, and its dominant-negative variant, MCUb. MCU is connected to the regulatory subunit MICU1 by the transmembrane protein EMRE. The MICU family also includes MICU1.1, MICU2, and MICU3. MICU proteins detect increases in Ca^2+^ levels through EF-hand domains, enabling the channel to open in response to elevated cytoplasmic Ca^2+^ levels. MCUR1 is a potential regulator of channel activity; however, its role is still a subject of debate.

The MCU complex is composed of the pore-forming subunit, MCU, its dominant-negative form, MCUb, the essential MCU regulator (EMRE), the mitochondrial Ca^2+^ uptake regulatory subunits (MICU1, MICU2, and MICU3), and potentially by the MCU regulator 1 (MCUR1) (Figure 1).

MCU is a highly conserved and ubiquitously expressed 40 KDa protein localized at the IMM. Structurally, MCU consists of two transmembrane domains separated by a short loop facing the intermembrane space (IMS). This loop is highly conserved due to the presence of negatively charged amino acids (“DIME” motif, composed of acidic residues), which are crucial for Ca^2+^ selectivity (Baughman et al., 2011; De Stefani et al., 2011). Consistent with its role of highly selective Ca^2+^ channel, downregulating MCU leads to a substantial reduction in mitCa2+ uptake without affecting the mitochondrial ΔΨ. Conversely, overexpression of MCU significantly enhances mitCa^2+^ uptake (De Stefani et al., 2011; Chaudhuri et al., 2013). Cryo-EM and X-ray diffraction structure analysis revealed that MCU arranges in a tetrameric architecture (Baradaran et al., 2018; Fan et al., 2018; Nguyen et al., 2018; Yoo et al., 2018), confuting a previously proposed pentameric structure of MCU (Oxenoid et al., 2016).

MCUb is an alternative isoform of MCU located in the IMM, where it forms hetero-oligomers with MCU (Raffaello et al., 2013). MCUb shares 50% sequence homology with MCU and has a similar structure: two transmembrane domains linked by a short loop. However, a crucial difference exists between these two pore-forming subunits. The MCUb protein sequence contains an amino acid substitution in the loop region (E256V) that neutralizes a negative charge, resulting in a significant reduction in the channel conductivity (Raffaello et al., 2013). In cells, overexpression of MCUb causes a reduction in mitCa^2+^ uptake when stimulated with a Ca^2+^ mobilizing agonist, while silencing MCUb strongly increases mtCa^2+^ uptake. This indicates that MCUb negatively affects Ca^2+^ entry through the MCU complex (Raffaello et al., 2013). The expression levels of MCUb vary significantly among different mammalian tissues, suggesting that the MCU/MCUb ratio might impact the physiological ability of mitochondria of specific tissues to uptake Ca^2+^. For instance, cardiomyocytes that display a low MCU/MCUb ratio are characterized by low MCU activity (Fieni et al., 2012), while skeletal muscle fibers, which instead exhibit high MCU activity (Fieni et al., 2012), are characterized by a high MCU/MCUb ratio. Interestingly, in heart MCUb incorporation in the complex is a stress-responsive mechanism to limit mitochondrial Ca^2+^ overload during cardiac injury (Lambert et al., 2019) and in skeletal muscle it is induced by caloric restriction, where it increases mitochondrial fatty acid utilization in a PDH-dependent mechanism (Huo et al., 2023). EMRE is a 10 kDa metazoan protein located in the IMM and composed of a single transmembrane domain (Sancak et al., 2013). This subunit connects the pore region to the regulatory subunits, as it is necessary for the interaction of MCU with MICU1 and MICU2 (Sancak et al., 2013). The function of the MCU complex critically relies on EMRE, as demonstrated by experiments on EMRE knockout cells. These experiments show that, in the absence of EMRE, mitCa^2+^ uptake is abolished, similar to the MCU knockout phenotype (Sancak et al., 2013). Also *in vivo*, EMRE has been shown to be required for mitochondrial calcium uniporter activity (Liu et al., 2020). The proteolytic regulation of EMRE, crucial for MCU complex function, is a finely tuned multi-step process that prevents the assembly of MCU-EMRE channels lacking gatekeeper subunits and, as a result, prevents mitochondrial mitCa^2+^ overload. (König et al., 2016).

The regulatory subunits MICU1, MICU2, and MICU3 are located at the IMS and are responsible for the sigmoidal increase of mitCa^2+^ in response to cytosolic Ca^2+^ levels. When cytosolic Ca^2+^ levels are low, mitCa^2+^ uptake is minimal, while it increases exponentially once the [Ca^2+^]_cyt_ reaches a certain threshold (Vecellio Reane et al., 2020) (Figure 1).

MICU1, the first subunit of the MCU complex to be identified in 2010, is a 54 KDa protein located in the IMS (Perocchi et al., 2010). The presence of two EF-hand Ca^2+^-binding domains at the N-terminal sequence enables MICU1 to regulate the activity of the MCU in a Ca^2+^-dependent manner. It was proposed that at low [Ca^2+^]_cyt_ levels, MICU1 keeps the channel closed to prevent continuous Ca^2+^ entry inside the mitochondrial matrix, which could lead to mitCa^2+^ overload if sustained over time. However, when a certain threshold of [Ca^2+^]_cyt_ is reached, MICU1 was proposed to act as a cooperative activator of MCU, explaining the exponential increase of mitCa^2+^ uptake (Mallilankaraman et al., 2012b; Csordás et al., 2013).

Two paralogs of MICU1 were later discovered: MICU2 and MICU3 (Plovanich et al., 2013). MICU2 exhibits a comparable expression pattern to MICU1, is also located in the IMS, and contains two EF-hand Ca^2+^-binding domains. MICU2 directly interacts with MICU1 and forms obligate heterodimers, which are stabilized by a disulfide bond. In various cell types, the loss of MICU1 also results in the depletion of MICU2 protein, suggesting that the protein stability of MICU2 is dependent on the presence of MICU1, in a mechanism that is not yet fully elucidated (Patron et al., 2014; Debattisti et al., 2019). Different models have been proposed to explain how the activity of the MCU channel is regulated by MICU1-MICU2 heterodimers. According to Patron et al., the channel is controlled by a gatekeeper mechanism, where MICU2 keeps the channel closed at resting conditions (Figure 1, left panel) (Patron et al., 2014). However, when the concentration of Ca^2+^ reaches a certain threshold, conformational changes in the dimers lead to the release of MICU2 inhibition, which results in an increased MICU1-mediated mitCa^2+^ uptake (Patron et al., 2014) (Figure 1, right panel). Kamer et al. proposed an on-off switch model for the channel activity. Both MICU1 and MICU2 act as gatekeepers and cooperatively bind Ca^2+^ with high affinity to lead to mitCa^2+^ uptake. In this model, in the absence of MICU2, MICU1 can keep the channel closed at low Ca^2+^ levels (Kamer et al., 2017). Another study suggests that the main role of MICU2 is to regulate the Ca^2+^ threshold of the MICU1-mediated channel activation (Payne et al., 2017).

It has been shown that skeletal muscle expresses a unique MCU complex. Indeed, this tissue expresses an alternative splicing variant of MICU1, known as MICU1.1 (Vecellio Reane et al., 2016). This variant has an extra exon that encodes a short sequence of four amino acids. When it forms dimers with MICU2, MICU1.1 activates the channel at a lower Ca^2+^ level compared to MICU1-MICU2 heterodimers. This is especially important in skeletal muscles, as it helps to maintain high ATP production (Vecellio Reane et al., 2016).

MICU3, the other paralog of MICU1, similarly to MICU2 is located in the IMS and contains two EF-hand Ca^2+^-binding domains (Plovanich et al., 2013). However, unlike MICU1 and MICU2 that are ubiquitous proteins, it is mainly expressed in the nervous system where it exclusively forms heterodimers with MICU1 (Plovanich et al., 2013; Patron et al., 2019). It acts as a positive channel regulator due to its reduced gatekeeping activity compared to MICU1. This ensures a more rapid opening of the channel in response to fast cytCa^2+^ increases, as it occurs in stimulated neuronal cells (Plovanich et al., 2013; Patron et al., 2019).

A possible regulator of the complex is MCUR1, a 35 kDa protein located in the IMM (Adlakha et al., 2019). This protein interacts with MCU and its silencing leads to reduced mitCa^2+^ uptake and ATP production (Mallilankaraman et al., 2012a). However, these effects were proposed to be mediated by its role as an assembly factor of the cytochrome-c oxidase (Paupe et al., 2015). In light of these conflicting results, further studies are needed to clarify the role of MCUR1 in the control of mitCa^2+^ homeostasis.

## 3 MitCa^2+^ buffering activity

As mentioned in the introduction, the presence of microdomains between mitochondria and ER/SR is an essential prerequisite for mitCa^2+^ uptake. Rapid changes in [Ca^2+^]_cyt_ occurring at these sites provide regulatory feedback on ER/SR Ca^2+^ channels (Rizzuto et al., 2012).

This buffering function is particularly relevant for Ins(1,4,5)P_3_Rs. The opening of these channels is inhibited by low and high [Ca^2+^]_cyt_, while intermediate [Ca^2+^]_cyt_ promotes their activity. An initial increase in [Ca^2+^]_cyt_ enables the opening of the Ins(1,4,5)P_3_Rs, thereby promoting the release of Ca^2+^ from the ER. MitCa^2+^ buffering plays a crucial role in sustaining and prolonging the release of Ca^2+^ by reducing the [Ca^2+^]_cyt_ near the Ins(1,4,5)P_3_Rs. This prevents the negative feedback associated with high [Ca^2+^]_cyt_ (Hajnóczky et al., 1999).

While the bell-shaped effect of [Ca^2+^]_cyt_ on Ins(1,4,5)P_3_Rs is commonly observed, there are some exceptions to this regulation with physiological relevance. One such exception is observed in rat cortical astrocytes, where the predominant isoform of the channel is Ins(1,4,5)P_3_R2. This isoform is positively regulated only by high [Ca^2+^]_cyt_, leading to limitations on mitochondrial involvement in the propagation of calcium waves (Boitier et al., 1999).

MitCa^2+^ buffering activity also holds particular significance in cardiac physiology. In neonatal cardiac cells, mitCa^2+^ uptake shapes the amplitude of Ca^2+^ peaks, as evidenced by genetic manipulation of MCU. Downregulation of MCU results in amplificated cytCa^2+^ peaks during spontaneous oscillations, while MCU overexpression has the opposite effect. Additionally, mitochondria buffer Ca^2+^ peaks by taking up Ca^2+^ released during systole and releasing it back into the cytosol during diastole (Drago et al., 2012).

MitCa^2+^ buffering capacity is also influenced by mitochondrial positioning in a defined subcellular domain. This is strongly evident in pancreatic acinar cells, where a “mitochondrial belt” separates the apical secretory pole from the basal pole containing the nucleus. Under normal conditions, this mitochondrial belt prevents the spreading of Ca^2+^ waves from the apical to the basal region. However, in pathological conditions where mitochondrial buffering capacity is overwhelmed, Ca^2+^ waves propagate to the basal region, leading to transcriptional events in the nucleus (Tinel et al., 1999; Sutton et al., 2003).

The role of mitochondrial positioning in regulating Ca^2+^ buffering is also observed in neurons. Specifically, mitochondria located at the synapse modulate cyt[Ca^2+^], strongly affecting neurotransmitter release (Billups and Forsythe, 2002; David and Barrett, 2003).

Overall, mitochondria possess an efficient Ca^2+^ buffering capacity that regulates cellular Ca^2+^ signals through the modulation of Ca^2+^ channels and their subcellular positioning.

## 4 MitCa^2+^ regulation of metabolism

**FIGURE 2:**
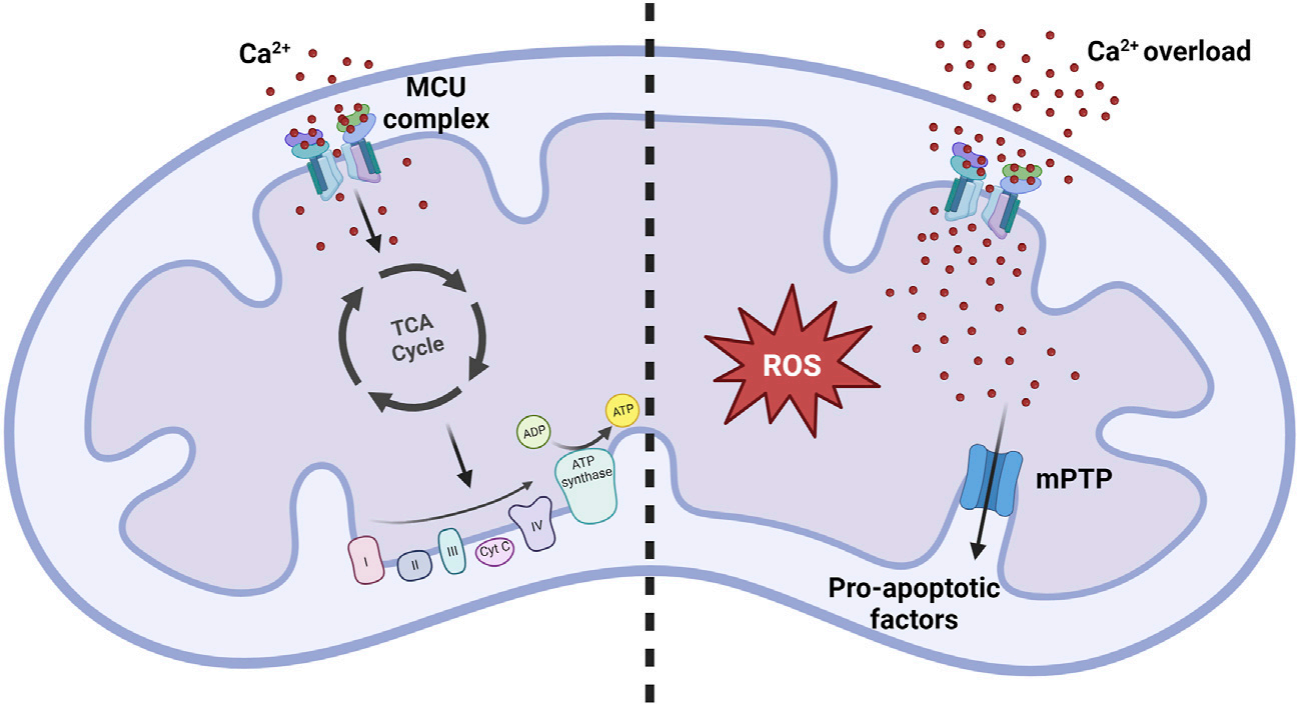
MitCa^2+^ uptake homeostasis. Under normal physiological conditions, the mitCa^2+^ enhances oxidative metabolism by increasing the activity of the Krebs cycle. Conditions of mitCa^2+^ overload promote the opening of the mPTP, resulting in the release of pro-apoptotic factors that ultimately lead to cell death. Simultaneously, excessive mitCa^2+^ uptake strongly promotes the formation of reactive oxygen species (ROS), contributing to the development of pathological conditions.

In the 1960s and 1970s, research carried out in Bristol revealed the pivotal role of mitCa^2+^ in regulating aerobic metabolism. Specifically, it was demonstrated that Ca^2+^ ions direclty activate four mitochondrial dehydrogenases, namely, α-ketoglutarate dehydrogenase (α-KGDH), isocitrate-dehydrogenases (IDH), FAD-linked glycerol phosphate dehydrogenase (GPDH), and indirectly, through its Ca^2+^-dependent phosphatase, pyruvate dehydrogenase (PDH) (Denton, 2009) (Figure 2, left panel). Significantly, the activation of these enzymes enhances the availability of NADH, promoting the electron flow through the respiratory chain complexes and, consequently, ATP synthesis. This is particularly relevant under increased ATP demand in stimulated cells.

- α-Ketoglutarate dehydrogenase (α-KGDH)

α-KGDH is an enzyme of the Krebs cycle responsible for converting α-ketoglutarate to succinyl-CoA. It comprises a complex of multiple enzymes with the core predominantly composed of dihydrolipoamide succinyl-transferase (E2) subunits, linked to 2-oxoglutarate decarboxylase (E1) and dihydrolipoamide dehydrogenase (E3) subunits (Yeaman, 1989). Ca^2+^ ions directly influence this enzyme by decreasing the K_m_ for α-ketoglutarate (McCormack and Denton, 1979).

- Isocitrate-dehydrogenases (IDH)

Another enzyme of the Krebs cycle directly activated by Ca^2+^ is IDH, which catalyzes the conversion of isocitrate in α-ketoglutarate. IDH consists of an octamer of three different subunits, with similar structure and molecular weight. Similar to α-KGDH, Ca^2+^ ions directly affect IDH by decreasing the K_m_ for its substrate isocitrate. However, for IDH, Ca^2+^ ion sensitivity is regulated by the ATP/ADP ratio, with increased sensitivity observed at lower ATP/ADP ratios (Denton et al., 1978; Rutter and Denton, 1988; 1989).

- FAD-linked glycerol phosphate dehydrogenase (GPDH)

This enzyme is located in the IMM and is part of the glycerol phosphate shuttle, along with the cytosolic NAD-dependent glycerol phosphate dehydrogenase. GPDH facilitates the transfer of reducing equivalents from cytosolic NADH to mitochondrial FADH_2_. Notably, this enzyme contains two EF-hand domains, in a region facing the IMS that are responsible for Ca^2+^ binding and increasing its activity (Wernette et al., 1981; MacDonald and Brown, 1996).

- Pyruvate dehydrogenase phosphate (PDP)

The pyruvate dehydrogenase complex (PDC) is a multi-enzyme complex that catalyzes the conversion of pyruvate to acetyl-CoA. The central core of the enzyme consists of dihydrolipoate acetyltransferase (E2) subunits, to which the pyruvate decarboxylase (E1) and the dihydrolipoate dehydrogenase (E3) subunits are attached (Hiromasa et al., 2004). Pyruvate dehydrogenase kinases (PDKs) catalyze reversible phosphorylation of three sites of the E1 subunits, inhibiting PDH activity. This inhibition can be reverted by the action of pyruvate dehydrogenase phosphatases (PDPs). In mammalian mitochondria, there are two isoforms of PDPs: PDP1 and PDP2. Importantly, only PDP1 is activated by Ca^2+^, leading to the dephosphorylation of PDH and its activation (Huang et al., 1998; Karpova et al., 2003).

It has also been proposed that, in addition to the four mitochondrial dehydrogenases, mitCa^2+^ can directly modulate the activity of the ATP synthase (Territo et al., 2000).

Furthermore, the aspartate/glutamate exchangers of the IMM (named SLC25A12 and SLC25A13) contain EF-hand Ca^2+^-binding sites exposed in the IMS. In response to a rise in cyt[Ca^2+^], metabolite transport is enhanced, ultimately stimulating ATP production (Lasorsa et al., 2003; Contreras et al., 2007).

## 5 MitCa^2+^ regulation of cell death

An excessive accumulation of Ca^2+^ ions inside the mitochondrial matrix, referred as to mitochondrial Ca^2+^ overload, is the primary trigger for the opening of the mitochondrial permeability transition pore (mPTP) (Figure 2, right panel). The opening of this channel leads to an unselective increase of the permeability of the IMM, allowing the exchange of small molecules across this membrane. This leads to a rapid collapse of the membrane potential, mitochondrial swelling, and subsequent release of pro-apoptotic mitochondrial components, including cytochrome *c*, ultimately culminating in cell death (Bernardi et al., 2022; Carraro and Bernardi, 2023).

MitCa^2+^ signals in apoptosis are tightly regulated by anti-apoptotic B cell lymphoma (BCL-2) proteins. These proteins modulate the ER-to-mitochondria Ca^2+^ transfer by enhancing the ER Ca^2+^ leak, thereby reducing the ER Ca^2+^ level. This reduction diminishes the transfer of Ca^2+^ to mitochondria upon extracellular stimuli (Foyouzi-Youssefi et al., 2000; Pinton et al., 2000; 2001; Palmer et al., 2004). In contrast, pro-apoptotic proteins exert the opposite effects (Scorrano et al., 2003). Another proposed mechanism involves the direct interaction and modulation of BCL-2 with Ca^2+^-releasing channels on the ER membrane, without affecting the ER Ca^2+^ level (Chen et al., 2004; Hanson et al., 2008; Rong et al., 2009). Overall, BCL-2 proteins can modulate the transfer of Ca^2+^ from the ER to the mitochondria by multiple mechanisms, thereby regulating mitCa^2+^ uptake in response to apoptotic stimuli.

MitCa^2+^ also plays a significan role in cell survival pathways. Specifically, the regulation of metabolism by mit[Ca^2+^] impacts autophagy. A decrease in mit[Ca^2+^], with the consequent reduction in the stimulation of aerobic metabolism, activates the AMP-activated protein kinase (AMPK), promoting autophagy. Notably, both the knockdown of Ins(1,4,5)P_3_Rs or the use of MCU blockers strongly increases autophagosome formation (Cárdenas et al., 2010). Consistent with these findings, silencing MCU, MICU1, or MCUR1 also serves as a potent activator of AMPK and, consequently, autophagy (Mallilankaraman et al., 2012a).

Overall, maintaining cellular homeostasis involves a complex balance between increasing MitCa^2+^ to meet cellular energy demands and minimizing the risk of mitochondrial calcium overload, a condition that promotes cell death.

## 6 Crosstalk of mitCa^2+^ and ROS production

Under normal physiological conditions, mitCa^2+^ uptake, by fueling oxidative metabolism, generates ROS signals. ROS are natural by-products of oxidative phosphorylation, and their concentration is tightly regulated by antioxidant molecules. Importantly, at low concentrations, ROS can act as a second messenger in the cell (Turrens, 2003). The physiological significance of mitCa^2+^ uptake in stimulating ROS production has been demonstrated in neurons, where it plays a critical role in the initiation of long-term potentiation (LTP), a fundamental form of synaptic plasticity. Specifically, MCU inhibition disrupt potentiation, despite the N-Methyl-d-aspartate (NMDA) receptor-mediated increase in cyt[Ca^2+^] (Kim et al., 2011).

However, in conditions of excessive mitCa^2+^ uptake, mitochondrial ROS production becomes detrimental (Feno et al., 2019). The increase in ROS production can be directly stimulated by the influence of Ca^2+^ on ROS-producing enzymes like α-KGDH and GPDH, or indirectly via nitric oxide synthase (NOS) activation, which generates NO causing an inhibition of complex IV (Görlach et al., 2015). Furthermore, an abundance of ROS is produced when the mPTP opens under conditions of mitCa^2+^ overload, by reverse electron transport (RET) following mitochondrial membrane depolarization (Biasutto et al., 2016).

ROS can cause damage to proteins, DNA, and lipids contributing to the development of diseases such as Duchenne muscular dystrophy (DMD) and cancer (Sies and Jones, 2020).

Loss of dystrophin in DMD leads to muscle membrane permeability and increased cyt[Ca^2+^]. One proposed mechanism suggests that the mitCa^2+^ overload resulting from the substantial rise in cyt[Ca^2+^] promotes ROS production, ultimately leading to muscle cell death through apoptotic pathways (Dubinin et al., 2020).

In the context of cancer, it has been demonstrated that Akt phosphorylation of MICU1 elevates basal mitCa^2+^ level and, consequently, ROS production, contributing to cancer progression (Marchi et al., 2019).

Taken together, these studies highlight that mitCa^2+^ serves as key regulator of ROS levels, and any dysregulation in mitCa^2+^ homeostasis may lead to excessive ROS production, fostering pathological conditions.

## 7 Conclusion

MitCa^2+^ uptake supports oxidative metabolism in response to increased cell energy demand, and its buffering capacity influences ER channels and cytosolic functions. Nevertheless, an excessive influx of mitochondrial mitCa^2+^ triggers the opening of the mitochondrial permeability transition pore (mPTP), ultimately culminating in cell death and fostering increased reactive oxygen species (ROS) production. Consequently, the maintenance of mitochondrial Ca^2+^ homeostasis is paramount for cellular functionality and survival..

## References

[B1] Adlakha J. Karamichali I. Sangwallek J. Deiss S. Bär K. Coles M. (2019). Characterization of MCU-binding proteins MCUR1 and CCDC90B - representatives of a protein family conserved in prokaryotes and eukaryotic organelles. Structure 27, 464–475. 10.1016/j.str.2018.11.004 30612859

[B2] Baradaran R. Wang C. Siliciano A. F. Long S. B. (2018). Cryo-EM structures of fungal and metazoan mitochondrial calcium uniporters. Nature 559, 580–584. 10.1038/s41586-018-0331-8 29995857 PMC6336196

[B3] Baughman J. M. Perocchi F. Girgis H. S. Plovanich M. Belcher-Timme C. A. Sancak Y. (2011). Integrative genomics identifies MCU as an essential component of the mitochondrial calcium uniporter. Nature 476, 341–345. 10.1038/nature10234 21685886 PMC3486726

[B4] Bernardi P. Carraro M. Lippe G. (2022). The mitochondrial permeability transition: recent progress and open questions. FEBS J. 289, 7051–7074. 10.1111/febs.16254 34710270 PMC9787756

[B5] Berridge M. J. Lipp P. Bootman M. D. (2000). The versatility and universality of calcium signalling. Nat. Rev. Mol. Cell. Biol. 1 (1), 11–21. 10.1038/35036035 11413485

[B6] Biasutto L. Azzolini M. Szabò I. Zoratti M. (2016). The mitochondrial permeability transition pore in AD 2016: an update. Biochim. Biophys. Acta 1863, 2515–2530. 10.1016/j.bbamcr.2016.02.012 26902508

[B7] Billups B. Forsythe I. D. (2002). Presynaptic mitochondrial calcium sequestration influences transmission at mammalian central synapses. J. Neurosci. 22, 5840–5847. 12122046 10.1523/JNEUROSCI.22-14-05840.2002 PMC6757942

[B8] Boitier E. Rea R. Duchen M. R. (1999). Mitochondria exert a negative feedback on the propagation of intracellular Ca2+ waves in rat cortical astrocytes. J. Cell Biol. 145, 795–808. 10.1083/jcb.145.4.795 10330407 PMC2133193

[B9] Cárdenas C. Miller R. A. Smith I. Bui T. Molgó J. Müller M. (2010). Essential regulation of cell bioenergetics by constitutive InsP3 receptor Ca2+ transfer to mitochondria. Cell 142, 270–283. 10.1016/j.cell.2010.06.007 20655468 PMC2911450

[B10] Carraro M. Bernardi P. (2023). The mitochondrial permeability transition pore in Ca2+ homeostasis. Cell Calcium 111, 102719. 10.1016/j.ceca.2023.102719 36963206

[B11] Chaudhuri D. Sancak Y. Mootha V. K. Clapham D. E. (2013). MCU encodes the pore conducting mitochondrial calcium currents. Elife 2, e00704. 10.7554/eLife.00704 23755363 PMC3673318

[B12] Chen R. Valencia I. Zhong F. McColl K. S. Roderick H. L. Bootman M. D. (2004). Bcl-2 functionally interacts with inositol 1,4,5-trisphosphate receptors to regulate calcium release from the ER in response to inositol 1,4,5-trisphosphate. J. Cell Biol. 166, 193–203. 10.1083/jcb.200309146 15263017 PMC2172311

[B13] Contreras L. Gomez-Puertas P. Iijima M. Kobayashi K. Saheki T. Satrústegui J. (2007). Ca2+ Activation kinetics of the two aspartate-glutamate mitochondrial carriers, aralar and citrin: role in the heart malate-aspartate NADH shuttle. J. Biol. Chem. 282, 7098–7106. 10.1074/jbc.M610491200 17213189

[B14] Csordás G. Golenár T. Seifert E. L. Kamer K. J. Sancak Y. Perocchi F. (2013). MICU1 controls both the threshold and cooperative activation of the mitochondrial Ca^2+^ uniporter. Cell Metab. 17, 976–987. 10.1016/j.cmet.2013.04.020 23747253 PMC3722067

[B15] Csordás G. Thomas A. P. Hajnóczky G. (1999). Quasi-synaptic calcium signal transmission between endoplasmic reticulum and mitochondria. EMBO J. 18, 96–108. 10.1093/emboj/18.1.96 9878054 PMC1171106

[B16] David G. Barrett E. F. (2003). Mitochondrial Ca2+ uptake prevents desynchronization of quantal release and minimizes depletion during repetitive stimulation of mouse motor nerve terminals. J. Physiol. 548, 425–438. 10.1113/jphysiol.2002.035196 12588898 PMC2342850

[B17] Debattisti V. Horn A. Singh R. Seifert E. L. Hogarth M. W. Mazala D. A. (2019). Dysregulation of mitochondrial Ca2+ uptake and sarcolemma repair underlie muscle weakness and wasting in patients and mice lacking MICU1. Cell Rep. 29, 1274–1286. 10.1016/j.celrep.2019.09.063 31665639 PMC7007691

[B18] Denton R. M. (2009). Regulation of mitochondrial dehydrogenases by calcium ions. Biochim. Biophys. Acta 1787, 1309–1316. 10.1016/j.bbabio.2009.01.005 19413950

[B19] Denton R. M. Richards D. A. Chin J. G. (1978). Calcium ions and the regulation of NAD+-linked isocitrate dehydrogenase from the mitochondria of rat heart and other tissues. Biochem. J. 176, 899–906. 10.1042/bj1760899 218557 PMC1186314

[B20] De Stefani D. Raffaello A. Teardo E. Szabò I. Rizzuto R. (2011). A forty-kilodalton protein of the inner membrane is the mitochondrial calcium uniporter. Nature 476, 336–340. 10.1038/nature10230 21685888 PMC4141877

[B21] Drago I. De Stefani D. Rizzuto R. Pozzan T. (2012). Mitochondrial Ca2+ uptake contributes to buffering cytoplasmic Ca2+ peaks in cardiomyocytes. Proc. Natl. Acad. Sci. U. S. A. 109, 12986–12991. 10.1073/pnas.1210718109 22822213 PMC3420165

[B22] Dubinin M. V. Talanov E. Y. Tenkov K. S. Starinets V. S. Mikheeva I. B. Sharapov M. G. (2020). Duchenne muscular dystrophy is associated with the inhibition of calcium uniport in mitochondria and an increased sensitivity of the organelles to the calcium-induced permeability transition. Biochim. Biophys. Acta Mol. Basis Dis. 1866, 165674. 10.1016/j.bbadis.2020.165674 31926263

[B23] Fan C. Fan M. Orlando B. J. Fastman N. M. Zhang J. Xu Y. (2018). X-ray and cryo-EM structures of the mitochondrial calcium uniporter. Nature 559, 575–579. 10.1038/s41586-018-0330-9 29995856 PMC6368340

[B24] Feno S. Butera G. Vecellio Reane D. Rizzuto R. Raffaello A. (2019). Crosstalk between calcium and ROS in pathophysiological conditions. Oxid. Med. Cell Longev. 2019, 9324018. 10.1155/2019/9324018 31178978 PMC6507098

[B25] Fieni F. Lee S. B. Jan Y. N. Kirichok Y. (2012). Activity of the mitochondrial calcium uniporter varies greatly between tissues. Nat. Commun. 3, 1317. 10.1038/ncomms2325 23271651 PMC3818247

[B26] Foyouzi-Youssefi R. Arnaudeau S. Borner C. Kelley W. L. Tschopp J. Lew D. P. (2000). Bcl-2 decreases the free Ca2+ concentration within the endoplasmic reticulum. Proc. Natl. Acad. Sci. U. S. A. 97, 5723–5728. 10.1073/pnas.97.11.5723 10823933 PMC18500

[B27] Görlach A. Bertram K. Hudecova S. Krizanova O. (2015). Calcium and ROS: a mutual interplay. Redox Biol. 6, 260–271. 10.1016/j.redox.2015.08.010 26296072 PMC4556774

[B28] Hajnóczky G. Hager R. Thomas A. P. (1999). Mitochondria suppress local feedback activation of inositol 1,4, 5-trisphosphate receptors by Ca2+. J. Biol. Chem. 274, 14157–14162. 10.1074/jbc.274.20.14157 10318833

[B29] Hanson C. J. Bootman M. D. Distelhorst C. W. Wojcikiewicz R. J. H. Roderick H. L. (2008). Bcl-2 suppresses Ca2+ release through inositol 1,4,5-trisphosphate receptors and inhibits Ca2+ uptake by mitochondria without affecting ER calcium store content. Cell Calcium 44, 324–338. 10.1016/j.ceca.2008.01.003 18407350

[B30] Hiromasa Y. Fujisawa T. Aso Y. Roche T. E. (2004). Organization of the cores of the mammalian pyruvate dehydrogenase complex formed by E2 and E2 plus the E3-binding protein and their capacities to bind the E1 and E3 components. J. Biol. Chem. 279, 6921–6933. 10.1074/jbc.M308172200 14638692

[B31] Huang B. Gudi R. Wu P. Harris R. A. Hamilton J. Popov K. M. (1998). Isoenzymes of pyruvate dehydrogenase phosphatase. DNA-derived amino acid sequences, expression, and regulation. J. Biol. Chem. 273, 17680–17688. 10.1074/jbc.273.28.17680 9651365

[B32] Huo J. Prasad V. Grimes K. M. Vanhoutte D. Blair N. S. Lin S.-C. (2023). MCUb is an inducible regulator of calcium-dependent mitochondrial metabolism and substrate utilization in muscle. Cell Rep. 42, 113465. 10.1016/j.celrep.2023.113465 37976157 PMC10842842

[B33] Kamer K. J. Grabarek Z. Mootha V. K. (2017). High-affinity cooperative Ca2+ binding by MICU1-MICU2 serves as an on-off switch for the uniporter. EMBO Rep. 18, 1397–1411. 10.15252/embr.201643748 28615291 PMC5538426

[B34] Karpova T. Danchuk S. Kolobova E. Popov K. M. (2003). Characterization of the isozymes of pyruvate dehydrogenase phosphatase: implications for the regulation of pyruvate dehydrogenase activity. Biochim. Biophys. Acta 1652, 126–135. 10.1016/j.bbapap.2003.08.010 14644048

[B35] Kim H. Y. Lee K. Y. Lu Y. Wang J. Cui L. Kim S. J. (2011). Mitochondrial Ca(2+) uptake is essential for synaptic plasticity in pain. J. Neurosci. 31, 12982–12991. 10.1523/JNEUROSCI.3093-11.2011 21900577 PMC3179262

[B36] König T. Tröder S. E. Bakka K. Korwitz A. Richter-Dennerlein R. Lampe P. A. (2016). The m -aaa protease associated with neurodegeneration limits MCU activity in mitochondria. Mol. Cell 64, 148–162. 10.1016/j.molcel.2016.08.020 27642048

[B37] Lambert J. P. Luongo T. S. Tomar D. Jadiya P. Gao E. Zhang X. (2019). MCUB regulates the molecular composition of the mitochondrial calcium uniporter channel to limit mitochondrial calcium overload during stress. Circulation 140, 1720–1733. 10.1161/CIRCULATIONAHA.118.037968 31533452 PMC6996560

[B38] Lasorsa F. M. Pinton P. Palmieri L. Fiermonte G. Rizzuto R. Palmieri F. (2003). Recombinant expression of the Ca(2+)-sensitive aspartate/glutamate carrier increases mitochondrial ATP production in agonist-stimulated Chinese hamster ovary cells. J. Biol. Chem. 278, 38686–38692. 10.1074/jbc.M304988200 12851387

[B39] Liu J. C. Syder N. C. Ghorashi N. S. Willingham T. B. Parks R. J. Sun J. (2020). EMRE is essential for mitochondrial calcium uniporter activity in a mouse model. JCI Insight 5, e134063. 10.1172/jci.insight.134063 32017711 PMC7101141

[B40] MacDonald M. J. Brown L. J. (1996). Calcium activation of mitochondrial glycerol phosphate dehydrogenase restudied. Arch. Biochem. Biophys. 326, 79–84. 10.1006/abbi.1996.0049 8579375

[B41] Mallilankaraman K. Cárdenas C. Doonan P. J. Chandramoorthy H. C. Irrinki K. M. Golenár T. (2012a). MCUR1 is an essential component of mitochondrial Ca2+ uptake that regulates cellular metabolism. Nat. Cell Biol. 14, 1336–1343. 10.1038/ncb2622 23178883 PMC3511605

[B42] Mallilankaraman K. Doonan P. Cárdenas C. Chandramoorthy H. C. Müller M. Miller R. (2012b). MICU1 is an essential gatekeeper for MCU-mediated mitochondrial Ca(2+) uptake that regulates cell survival. Cell 151, 630–644. 10.1016/j.cell.2012.10.011 23101630 PMC3486697

[B43] Marchi S. Corricelli M. Branchini A. Vitto V. A. M. Missiroli S. Morciano G. (2019). Akt-mediated phosphorylation of MICU1 regulates mitochondrial Ca2+ levels and tumor growth. EMBO J. 38, e99435. 10.15252/embj.201899435 30504268 PMC6331721

[B44] McCormack J. G. Denton R. M. (1979). The effects of calcium ions and adenine nucleotides on the activity of pig heart 2-oxoglutarate dehydrogenase complex. Biochem. J. 180, 533–544. 10.1042/bj1800533 39549 PMC1161091

[B45] Mitchell P. Moyle J. (1967). Chemiosmotic hypothesis of oxidative phosphorylation. Nature 213, 137–139. 10.1038/213137a0 4291593

[B46] Nguyen N. X. Armache J.-P. Lee C. Yang Y. Zeng W. Mootha V. K. (2018). Cryo-EM structure of a fungal mitochondrial calcium uniporter. Nature 559, 570–574. 10.1038/s41586-018-0333-6 29995855 PMC6063787

[B47] Oxenoid K. Dong Y. Cao C. Cui T. Sancak Y. Markhard A. L. (2016). Architecture of the mitochondrial calcium uniporter. Nature 533, 269–273. 10.1038/nature17656 27135929 PMC4874835

[B48] Palmer A. E. Jin C. Reed J. C. Tsien R. Y. (2004). Bcl-2-mediated alterations in endoplasmic reticulum Ca2+ analyzed with an improved genetically encoded fluorescent sensor. Proc. Natl. Acad. Sci. U. S. A. 101, 17404–17409. 10.1073/pnas.0408030101 15585581 PMC535104

[B49] Patron M. Checchetto V. Raffaello A. Teardo E. Vecellio Reane D. Mantoan M. (2014). MICU1 and MICU2 finely tune the mitochondrial Ca2+ uniporter by exerting opposite effects on MCU activity. Mol. Cell 53, 726–737. 10.1016/j.molcel.2014.01.013 24560927 PMC3988891

[B50] Patron M. Granatiero V. Espino J. Rizzuto R. De Stefani D. (2019). MICU3 is a tissue-specific enhancer of mitochondrial calcium uptake. Cell Death Differ. 26, 179–195. 10.1038/s41418-018-0113-8 29725115 PMC6124646

[B51] Paupe V. Prudent J. Dassa E. P. Rendon O. Z. Shoubridge E. A. (2015). CCDC90A (MCUR1) is a cytochrome c oxidase assembly factor and not a regulator of the mitochondrial calcium uniporter. Cell Metab. 21, 109–116. 10.1016/j.cmet.2014.12.004 25565209

[B52] Payne R. Hoff H. Roskowski A. Foskett J. K. (2017). MICU2 restricts spatial crosstalk between InsP3R and MCU channels by regulating threshold and gain of MICU1-mediated inhibition and activation of MCU. Cell Rep. 21, 3141–3154. 10.1016/j.celrep.2017.11.064 29241542 PMC5734103

[B53] Perocchi F. Gohil V. M. Girgis H. S. Bao X. R. McCombs J. E. Palmer A. E. (2010). MICU1 encodes a mitochondrial EF hand protein required for Ca(2+) uptake. Nature 467, 291–296. 10.1038/nature09358 20693986 PMC2977980

[B54] Pinton P. Ferrari D. Magalhães P. Schulze-Osthoff K. Di Virgilio F. Pozzan T. (2000). Reduced loading of intracellular Ca(2+) stores and downregulation of capacitative Ca(2+) influx in Bcl-2-overexpressing cells. J. Cell Biol. 148, 857–862. 10.1083/jcb.148.5.857 10704437 PMC2174537

[B55] Pinton P. Ferrari D. Rapizzi E. Di Virgilio F. Pozzan T. Rizzuto R. (2001). The Ca2+ concentration of the endoplasmic reticulum is a key determinant of ceramide-induced apoptosis: significance for the molecular mechanism of Bcl-2 action. EMBO J. 20, 2690–2701. 10.1093/emboj/20.11.2690 11387204 PMC125256

[B56] Plovanich M. Bogorad R. L. Sancak Y. Kamer K. J. Strittmatter L. Li A. A. (2013). MICU2, a paralog of MICU1, resides within the mitochondrial uniporter complex to regulate calcium handling. PLoS One 8, e55785. 10.1371/journal.pone.0055785 23409044 PMC3567112

[B57] Raffaello A. De Stefani D. Sabbadin D. Teardo E. Merli G. Picard A. (2013). The mitochondrial calcium uniporter is a multimer that can include a dominant-negative pore-forming subunit. EMBO J. 32, 2362–2376. 10.1038/emboj.2013.157 23900286 PMC3771344

[B58] Rizzuto R. De Stefani D. Raffaello A. Mammucari C. (2012). Mitochondria as sensors and regulators of calcium signalling. Nat. Rev. Mol. Cell Biol. 13, 566–578. 10.1038/nrm3412 22850819

[B59] Rizzuto R. Pinton P. Carrington W. Fay F. S. Fogarty K. E. Lifshitz L. M. (1998). Close contacts with the endoplasmic reticulum as determinants of mitochondrial Ca2+ responses. Science 280, 1763–1766. 10.1126/science.280.5370.1763 9624056

[B60] Rong Y.-P. Bultynck G. Aromolaran A. S. Zhong F. Parys J. B. De Smedt H. (2009). The BH4 domain of Bcl-2 inhibits ER calcium release and apoptosis by binding the regulatory and coupling domain of the IP3 receptor. Proc. Natl. Acad. Sci. U. S. A. 106, 14397–14402. 10.1073/pnas.0907555106 19706527 PMC2728114

[B61] Rutter G. A. Denton R. M. (1988). Regulation of NAD+-linked isocitrate dehydrogenase and 2-oxoglutarate dehydrogenase by Ca2+ ions within toluene-permeabilized rat heart mitochondria. Interactions with regulation by adenine nucleotides and NADH/NAD+ ratios. Biochem. J. 252, 181–189. 10.1042/bj2520181 3421900 PMC1149122

[B62] Rutter G. A. Denton R. M. (1989). The binding of Ca2+ ions to pig heart NAD+-isocitrate dehydrogenase and the 2-oxoglutarate dehydrogenase complex. Biochem. J. 263, 453–462. 10.1042/bj2630453 2597117 PMC1133450

[B63] Sancak Y. Markhard A. L. L. Kitami T. Kovács-Bogdán E. Kamer K. J. J. Udeshi N. D. D. (2013). EMRE is an essential component of the mitochondrial calcium uniporter complex. Science 342, 1379–1382. 10.1126/science.1242993 24231807 PMC4091629

[B64] Scorrano L. Oakes S. A. Opferman J. T. Cheng E. H. Sorcinelli M. D. Pozzan T. (2003). BAX and BAK regulation of endoplasmic reticulum Ca2+: a control point for apoptosis. Science 300, 135–139. 10.1126/science.1081208 12624178

[B65] Sies H. Jones D. P. (2020). Reactive oxygen species (ROS) as pleiotropic physiological signalling agents. Nat. Rev. Mol. Cell Biol. 21, 363–383. 10.1038/s41580-020-0230-3 32231263

[B66] Sutton R. Criddle D. Raraty M. G. T. Tepikin A. Neoptolemos J. P. Petersen O. H. (2003). Signal transduction, calcium and acute pancreatitis. Pancreatology 3, 497–505. 10.1159/000075581 14673201

[B67] Szalai G. Csordás G. Hantash B. M. Thomas A. P. Hajnóczky G. (2000). Calcium signal transmission between ryanodine receptors and mitochondria. J. Biol. Chem. 275, 15305–15313. 10.1074/jbc.275.20.15305 10809765

[B68] Territo P. R. Mootha V. K. French S. A. Balaban R. S. (2000). Ca(2+) activation of heart mitochondrial oxidative phosphorylation: role of the F(0)/F(1)-ATPase. Am. J. Physiol. Cell Physiol. 278, C423–C435. 10.1152/ajpcell.2000.278.2.C423 10666039

[B69] Tinel H. Cancela J. M. Mogami H. Gerasimenko J. V. Gerasimenko O. V. Tepikin A. V. (1999). Active mitochondria surrounding the pancreatic acinar granule region prevent spreading of inositol trisphosphate-evoked local cytosolic Ca(2+) signals. EMBO J. 18, 4999–5008. 10.1093/emboj/18.18.4999 10487752 PMC1171571

[B70] Turrens J. F. (2003). Mitochondrial formation of reactive oxygen species. J. Physiol. 552, 335–344. 10.1113/jphysiol.2003.049478 14561818 PMC2343396

[B71] Vecellio Reane D. Rizzuto R. Raffaello A. (2020). The ER-mitochondria tether at the hub of Ca2+ signaling. Curr. Opin. Physiol. 17, 261–268. 10.1016/j.cophys.2020.08.013

[B72] Vecellio Reane D. Vallese F. Checchetto V. Acquasaliente L. Butera G. De Filippis V. (2016). A MICU1 splice variant confers high sensitivity to the mitochondrial Ca2+ uptake machinery of skeletal muscle. Mol. Cell 64, 760–773. 10.1016/j.molcel.2016.10.001 27818145

[B73] Wernette M. E. Ochs R. S. Lardy H. A. (1981). Ca2+ stimulation of rat liver mitochondrial glycerophosphate dehydrogenase. J. Biol. Chem. 256, 12767–12771. 10.1016/s0021-9258(18)42961-4 6796576

[B74] Yeaman S. J. (1989). The 2-oxo acid dehydrogenase complexes: recent advances. Biochem. J. 257, 625–632. 10.1042/bj2570625 2649080 PMC1135633

[B75] Yoo J. Wu M. Yin Y. Herzik M. A. Lander G. C. Lee S.-Y. (2018). Cryo-EM structure of a mitochondrial calcium uniporter. Science 361, 506–511. 10.1126/science.aar4056 29954988 PMC6155975

